# Right Femoral Pseudoaneurysm Versus Inguinal Abscess: Progressive Inguinal Mass Following Blunt Trauma

**DOI:** 10.7759/cureus.92305

**Published:** 2025-09-14

**Authors:** Emily Cushman, Danijela Lonco, James White, Rebecca Perry

**Affiliations:** 1 Emergency Medicine, Kettering Health, Dayton, USA

**Keywords:** blunt trauma, groin abscess, imaging, inguinal mass, pseudoaneurysm

## Abstract

When evaluating a patient with inguinal swelling following blunt trauma, maintaining a broad differential diagnosis is crucial, especially when laboratory and imaging findings do not correlate with the patient’s history. An important consideration is the choice of imaging modality to assess progressively worsening inguinal pain and swelling. A 21-year-old healthy female presented to the ED with a progressively enlarging mass in the right inguinal region following traumatic injury. She had undergone two prior evaluations by an OB-GYN, with recommendations for acetaminophen and NSAIDs, which provided no relief. The mass had increased in size relative to her discomfort, causing difficulty ambulating. On initial presentation, she was afebrile but tachycardic. Physical examination revealed significant right-sided vaginal edema extending throughout the inguinal region without overlying integumentary changes, abrasion, laceration, or ecchymosis. Laboratory evaluation was remarkable only for leukocytosis. Advanced imaging identified a right inguinal pseudoaneurysm of indeterminate vascular origin with a surrounding hematoma measuring 4.3 × 3.7 cm. The patient was subsequently hospitalized for vascular surgery consultation. Vascular ultrasonography was performed to further characterize the lesion, revealing a vascularized region in the right groin measuring 2.07 × 1.17 cm, although a pseudoaneurysm could not be ruled out. Ultimately, the patient was taken to the operating room, where an abscess with purulent drainage was identified. Sonographic imaging may have limitations in differentiating a hematoma or pseudoaneurysm from an abscess due to similar anechoic to hypoechoic findings. This patient presented with a progressively worsening inguinal mass unresponsive to acetaminophen and ibuprofen, with overlying erythema. While ultrasonography is a logical initial imaging modality to rule out a simple abscess, the presence of Doppler flow should prompt consideration of further imaging with CT angiography. CT angiography provides a detailed evaluation of vascular involvement and associated pathology, and it aids in treatment planning, including decisions regarding medical management versus surgical intervention.

## Introduction

Inguinal masses can have a multitude of causes, ranging from relatively benign conditions such as lipomas or cysts to more concerning etiologies like pseudoaneurysms, malignancy, or abscesses. Because of the wide spectrum of possible causes and presentations, appropriate evaluation and diagnostic imaging are essential [1]. Imaging that helps determine the likely origin of the mass is particularly important. Intrapelvic versus extrapelvic masses require distinct considerations for further evaluation and management [2]. CT imaging should be considered, given the difficulty in differentiating hematomas, pseudoaneurysms, and abscesses on ultrasound [3,4].

Pseudoaneurysms are uncommon in young individuals, with the most frequent causes being recent endovascular procedures; however, blunt trauma can also result in vascular injury. Here, we present a case of an abscess that mimicked the features of a pseudoaneurysm and was initially diagnosed as such on imaging following blunt trauma to the groin.

## Case presentation

A 21-year-old previously healthy female presented to our ED with complaints of a progressively worsening mass in her right groin. She reported first noticing the mass after an incidental blunt trauma to the groin. She had previously been evaluated by her OB-GYN, who recommended acetaminophen and NSAIDs, which provided no relief. The increasing discomfort and eventual difficulty ambulating due to pain prompted her arrival in the ED. She had no prior history of a mass in the inguinal region.

**Figure 1:**
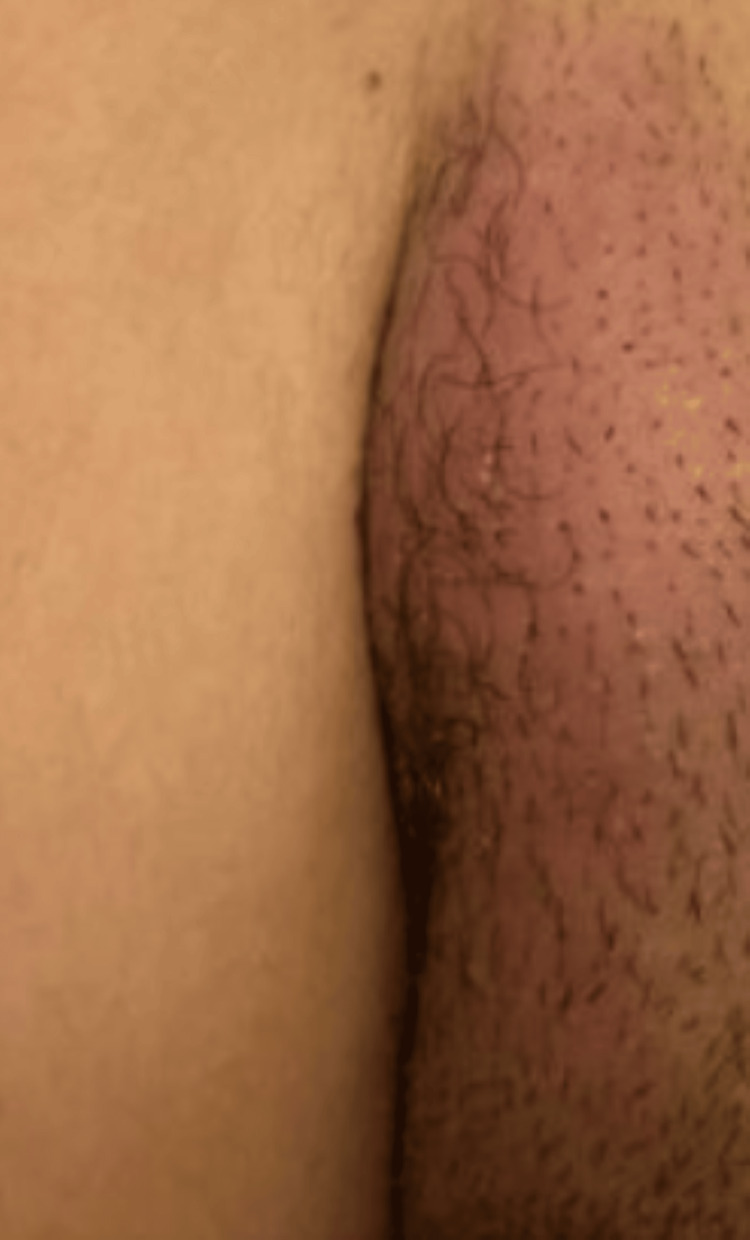
Physical exam findings in the ED Right inguinal fold mass with mild overlying erythema. No obvious signs of trauma.

On arrival, vital signs revealed tachycardia and fever. Physical examination demonstrated significant right-sided vaginal edema extending throughout the inguinal region without overlying integumentary changes, abrasion, laceration, or ecchymosis. A mass measuring approximately 3 × 4 cm was noted (Figure 1). The mass was nonpulsatile.

**Figure 2:**
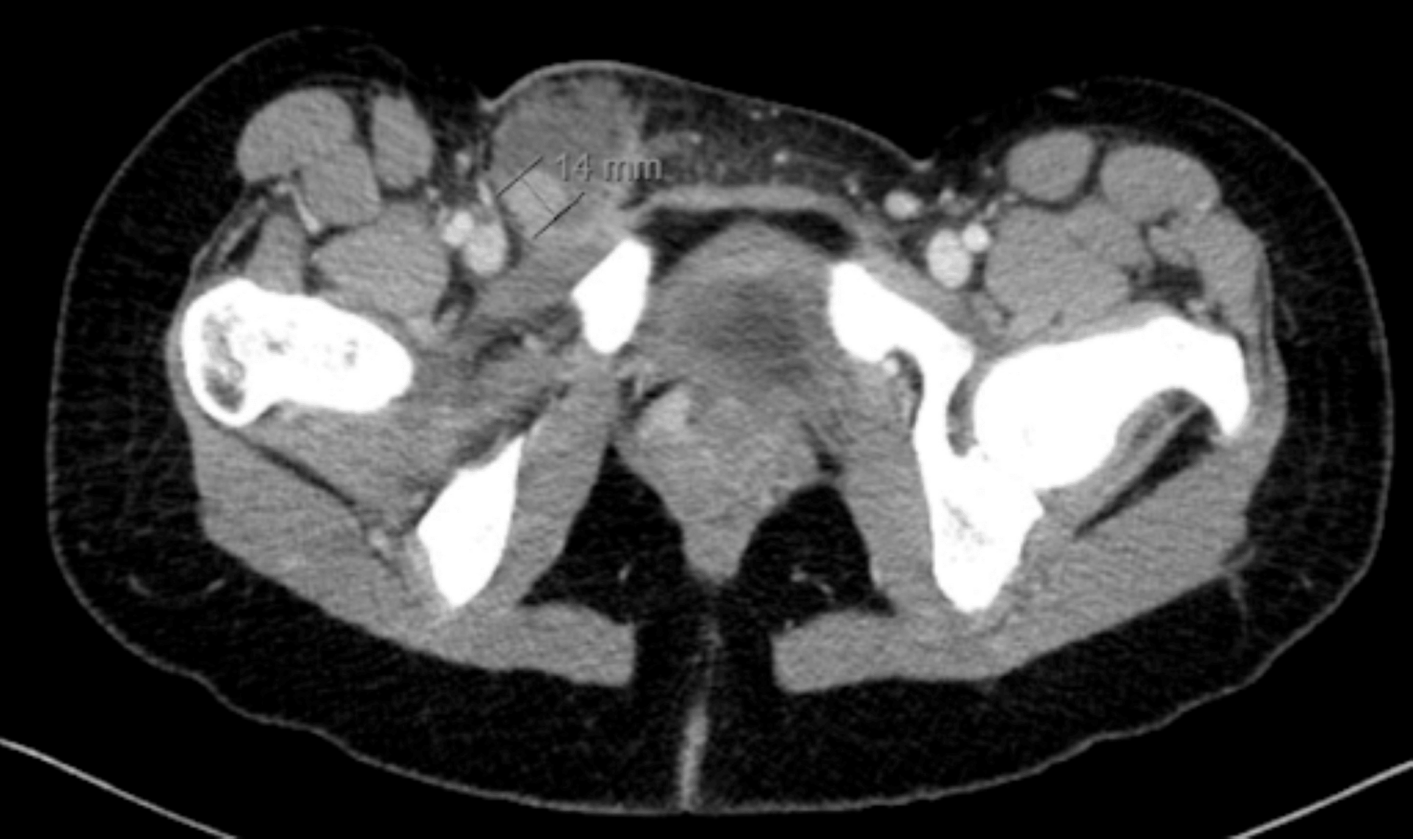
CT pelvis, static image Findings suggest a pseudoaneurysm with a surrounding hematoma. No obvious signs of localized fat stranding.

Laboratory studies were obtained and were notable only for a leukocytosis of 18. Initial imaging included a CT of the pelvis, which demonstrated a right inguinal pseudoaneurysm of indeterminate origin with a surrounding hematoma measuring 4.3 × 3.7 cm (Figure 2).

**Figure 3:**
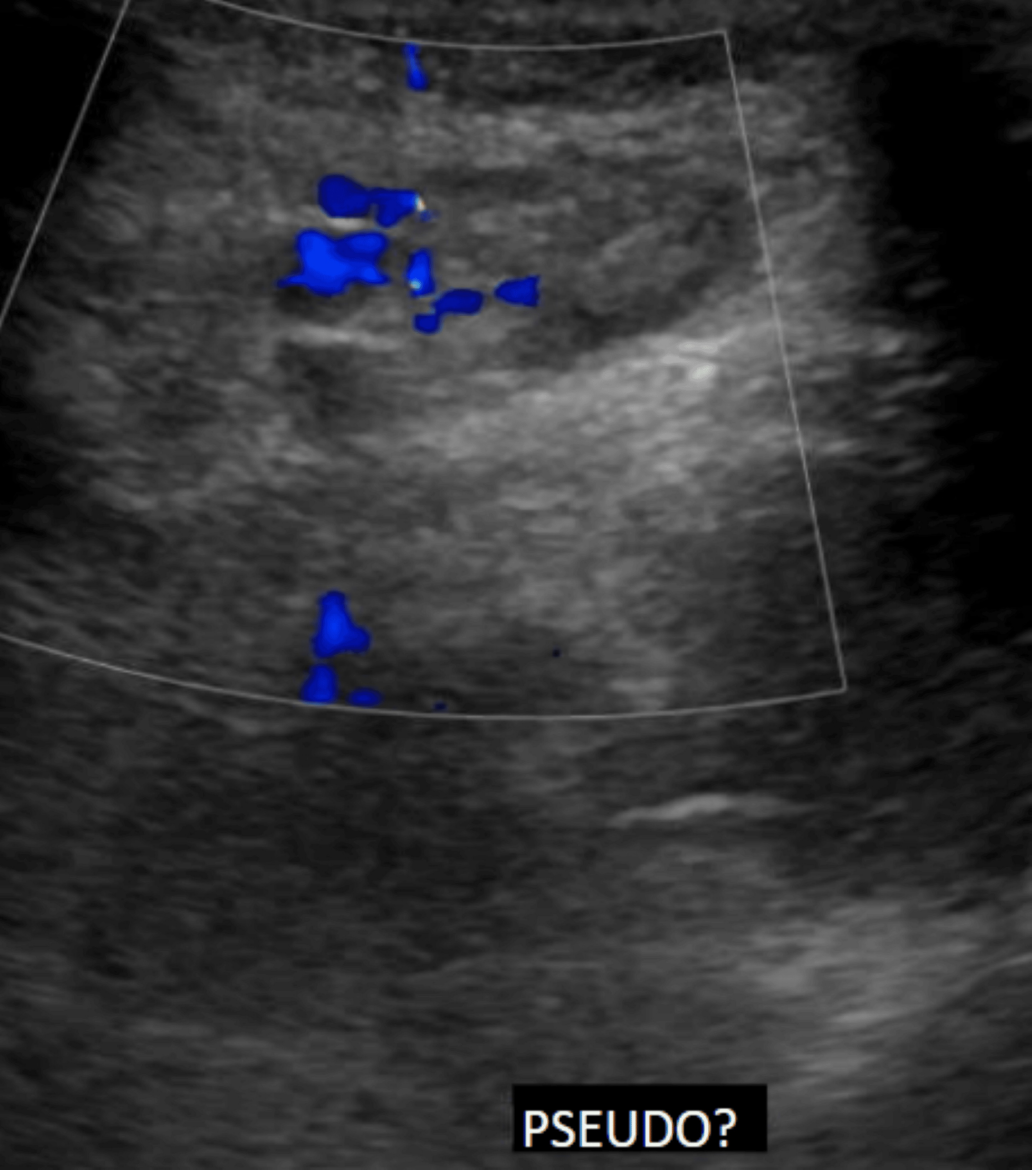
Vascular ultrasound of the right inguinal mass, static image Doppler flow is noted within the mass. Imaging is indeterminate, and a pseudoaneurysm cannot be ruled out.

Vascular surgery was consulted, and the patient was admitted for further evaluation and management. A vascular ultrasound performed by the vascular surgeon revealed a vascularized region measuring 2.07 × 1.17 cm, with an inability to rule out a pseudoaneurysm (Figure 3). The patient was subsequently taken to the operating room for further intervention.

During incision and local exploration, an abscess was identified in the right inguinal region. The abscess was incised and drained, with purulent material noted. Cultures were obtained, and the area was thoroughly irrigated. Cultures grew *Streptococcus pyogenes*. The patient was started on appropriate intravenous antibiotics, later transitioned to oral antibiotics, and was eventually discharged home with outpatient follow-up.

## Discussion

Inguinal masses can arise from a variety of pathologies, with emphasis on differentiating localized soft tissue masses or infections from deeper underlying pathology [1]. Typically, femoral pseudoaneurysms occur secondary to recent vascular access; however, they can also result from blunt trauma, as in this patient, and may progressively enlarge, potentially leading to rupture [5]. The primary focus in the ED is to establish the most likely diagnosis and determine appropriate next steps for treatment or management. An important aspect of this process is selecting the most appropriate diagnostic imaging or determining whether imaging is indicated at that time. The two most common imaging modalities obtained in the ED are CT and ultrasound, either point of care or formal.

When selecting imaging, the patient’s presentation, history, and physical examination findings must be considered. Suspected soft tissue masses are often initially evaluated via point-of-care ultrasound to assess for drainable fluid collections beneath the skin surface [6,7]. However, sonographic imaging can be misleading. Ultrasound may have difficulty differentiating a pseudoaneurysm from an abscess or hematoma due to similar anechoic to hypoechoic appearances, making clinical correlation essential [4].

In this case, the patient presented with a progressively enlarging inguinal mass, unresponsive to acetaminophen and ibuprofen, with mild overlying erythema. Ultrasound is a reasonable initial modality to rule out a simple abscess and minimize unnecessary radiation exposure; however, if Doppler flow is noted, further imaging with CT angiography should be considered. CT angiography provides detailed information regarding vascular involvement and other pathology [4], and it also facilitates treatment planning, whether medical management or surgical intervention. Despite its utility, imaging is not always conclusive, as demonstrated in this case.

For most abscesses, point-of-care ultrasound is appropriate for assessing the size of the fluid collection prior to incision and drainage. Clinical symptoms and laboratory findings can also aid in diagnosis, as pyogenic inguinal abscesses may present with fever and leukocytosis [2]. However, for suspected inguinal abscesses, ultrasound alone is insufficient. CT should be performed to determine whether the abscess is intrapelvic or extrapelvic and to evaluate for communication or tract formation [1,2]. Determining the abscess origin helps guide appropriate antibiotic therapy; intrapelvic abscesses require coverage for anaerobic organisms [2]. Doppler ultrasound may help identify blood flow, but it can be inconclusive, as seen in this case, and its accuracy depends on the operator’s skill and probe positioning.

Not all patient presentations have histories that clearly align with the final diagnosis. In this case, the patient presented after blunt trauma to the groin with worsening symptoms suggestive of possible hematoma expansion or vascular injury. Leukocytosis and fever may have been attributable to a stress response from trauma as well as inflammation from a developing infection or abscess [2]. Ultimately, the patient required surgical intervention, either for vascular repair or incision and drainage of an abscess.

## Conclusions

Due to the complexity of the pelvic region and the wide range of potential pathologies, CT is the optimal diagnostic imaging modality for evaluating inguinal masses, particularly in patients with concerning presentations. Point-of-care ultrasound can be useful for initial differentiation of solid versus cystic or fluid-filled masses but should not serve as the definitive diagnostic tool for concerning inguinal masses.
